# On propagation in networks, promising models beyond network diffusion to describe degenerative brain diseases and traumatic brain injuries

**DOI:** 10.3389/fphar.2024.1321171

**Published:** 2024-02-26

**Authors:** Davide Vergni, Paola Stolfi, Annalisa Pascarella

**Affiliations:** Institute for Applied Mathematics (IAC), National Research Council (CNR), Rome, Italy

**Keywords:** traumatic brain injury, connectome, complex network, network diffusion, propagation on network

## Abstract

**Introduction:** Connections among neurons form one of the most amazing and effective network in nature. At higher level, also the functional structures of the brain is organized as a network. It is therefore natural to use modern techniques of network analysis to describe the structures of networks in the brain. Many studies have been conducted in this area, showing that the structure of the neuronal network is complex, with a small-world topology, modularity and the presence of hubs. Other studies have been conducted to investigate the dynamical processes occurring in brain networks, analyzing local and large-scale network dynamics. Recently, network diffusion dynamics have been proposed as a model for the progression of brain degenerative diseases and for traumatic brain injuries.

**Methods:** In this paper, the dynamics of network diffusion is re-examined and reaction-diffusion models on networks is introduced in order to better describe the degenerative dynamics in the brain.

**Results:** Numerical simulations of the dynamics of injuries in the brain connectome are presented. Different choices of reaction term and initial condition provide very different phenomenologies, showing how network propagation models are highly flexible.

**Discussion:** The uniqueness of this research lies in the fact that it is the first time that reaction-diffusion dynamics have been applied to the connectome to model the evolution of neurodegenerative diseases or traumatic brain injury. In addition, the generality of these models allows the introduction of non-constant diffusion and different reaction terms with non-constant parameters, allowing a more precise definition of the pathology to be studied.

## 1 Introduction

Dynamics occurring in the connectome are crucial because they underlie the brain’s ability to process information, learn, and adapt to changing environments (Avena-Koenigsberger et al., 2018). Understanding these dynamics is essential for unraveling the neural basis of cognition, behavior, and, medically, neurological issues. From this perspective, any study that leads to progress in the understanding of the mechanisms associated with neurological deficits is potentially a step forward in the development of methodologies that can help recover brain health (Raj et al., 2012; Poudel et al., 2020).

From the introduction of the term connectome (Sporns et al., 2005), when its exact structure was largely unknown, until today, several research studies have been done to study the very complex network of the connectome (Bullmore and Sporns, 2009) and the dynamic processes occurring in it (Avena-Koenigsberger et al., 2018).

In particular, human connectome dynamics occur in multiple time scales, ranging from milliseconds to years, and different types of measuring equipment are used to capture them (Mitra, 2007). These different time scales reveal various aspects of brain functions and behaviors. The shortest time scales are related to rapid neural processing and information exchange within functional brain networks. Neurotransmission and synaptic communication play a vital role in this fast-paced activity. Electroencephalography (EEG) and magnetoencephalography (MEG) are elective techniques for capturing these rapid electrical brain signals. At higher time scales, from seconds to minutes, the dynamics of the connectome are related to cognitive processes and functional connectivity changes during specific tasks. Functional MRI (fMRI) is commonly used to study these changes. For example, during a memory task, certain brain regions may exhibit increased functional activity, indicating their involvement in the memory network (Murphy et al., 2020). From minutes to hours, the dynamics of the connectome are related to resting-state fluctuations in functional connectivity (Smitha et al., 2017). Resting-state fMRI is used to study intrinsic brain activity while an individual is not performing any specific task. Examples of processes that occur at higher time scales, from days to years, are learning, memory consolidation processes, brain development, and cognitive decline. In particular, the processes which we are interested in, traumatic brain injuries and degenerative brain dynamics, occur on these time scales. For these kinds of diseases, it is of paramount importance to integrate the functional information with that arising from the study of the structural connectome, which represents the anatomical connections between different brain regions. Diffusion tensor imaging (DTI) and diffusion-weighted imaging (DWI) are the main commonly used MRI techniques to create a structural connectome. We chose to use the connectome created from DTI and DWI data because there is evidence that it is involved in the propagation of neurological diseases (Torok et al., 2018; Weickenmeier et al., 2019; Wilson et al., 2023). However, it is important to emphasize that the methodology presented in this work is independent of the type of connectome one decides to use (whether based on functionality, proximity, synaptic connections, or some other structure of brain physiology); the most appropriate network must be chosen to accurately describe the propagation of a given neural pathology.

A growing number of works on degenerative brain diseases (Raj et al., 2012; Raj et al., 2015; Pandya et al., 2019) and traumatic brain injuries (Poudel et al., 2020) use network diffusion as a descriptive and predictive dynamical model.

The network diffusion process, also known as the heat diffusion process, is becoming increasingly important in all those applications where some kind of network dynamics needs to be modeled. The fields of application are the most varied, from machine learning (see, for example, (Hofmann et al., 2008) and the recent (Stolfi et al., 2023)) to network biology (see (Carlin et al., 2017) and reference therein) and from the propagation of epidemics (see, for example, (Masuda, 2010)) to degenerative diseases of the brain (Raj et al., 2012, 2015; Pandya et al., 2019; Poudel et al., 2020), which is where our attention will be focused.

However, with regard to the progression of brain degenerative diseases or brain injuries, network diffusion is not the best modeling description. Diffusion is introduced for the description of the random movements of particles or substances through a medium due to thermal agitation, and the diffusion equation preserves the amount of spreading particles or substances. This condition, in the case of a progressive disease or increasing brain damage, cannot be satisfied. We cannot assume that brain damage will be preserved; most probably, it may increase or decrease. Rather than a diffusing substance, the progressive worsening of dementia, for example, might be better characterized by the progression of damage, which, in modeling terms, can be described by a propagating front associated with reaction–diffusion equations.

In neurological applications, reaction–diffusion models on networks have been used very little and only recently. When they are used, they are not directly coupled to the connectome (Andrade-Restrepo et al., 2021) or diffusion on the lattice is used (Schmitt et al., 2022).

In the present work, we illustrate a simple methodology to describe propagation dynamics on the connectome using reaction–diffusion dynamics on graphs. We apply the model to a simple connectome, and we show the differences between a simple network diffusion model and a propagation model.

## 2 Materials and methods

In this section, we briefly recall the theory of network diffusion models (Section 2.1) and network reaction–diffusion models (Section 2.2). Then, we analyze some reaction terms that can be used within the latter models (Section 2.3), and finally, we proceed with the description of the dataset used to apply the mentioned network dynamics models on the connectome.

### 2.1 Diffusion on networks

The connectome is usually described (Bullmore and Bassett, 2011) by an undirected and unweighted graph, *G* = (*V*, *E*), where *V* is the set of vertices (or nodes) of the graph (we consider a finite number, *N*, of vertices) and *E* is the set of edges (or links) connecting the vertices. The graph is determined by its adjacency matrix *A*_*ij*_ (Bollobás, 1998), which is given by

$$A_{ij}=\left\{\begin{array}{cc} 1 & \text{if }\;\left(i,j\right)\in E\\ 0 & \text{if }\;\left(i,j\right)\notin E \end{array}\right.$$ (1)

An important quantity associated with each node, *i*, is its degree, *k*_*i*_, defined as the number of nearest neighbors of *i*. This quantity can be computed using the adjacency matrix *k*_*i*_ = *∑*_*j*_ *A*_*ij*_ as defined in Eq. 1.

Defining the concentration of substances contained in each node, *θ*_*i*_(*t*), we can describe the diffusion dynamics on a graph by considering the flow, with a diffusion rate *w*, of that quantity from the various edges of the graph. The increase in the concentration in each node in time d*t* can be computed as follows:

$$\theta_{i}\left(t+dt\right)-\theta_{i}\left(t\right)=w\left[\left(\underset{j}{\sum }A_{ij}\theta_{j}\left(t\right)\right)-k_{i}\theta_{i}\left(t\right)\right]dt.$$ (2)

The first term on the right side of Eq. 2 accounts for the inflow of substances coming from the neighbors of node *i*, while the second term accounts for the outflow of substances from node *i* to its neighbors. Equation 2 can be rewritten as follows:

$$\theta_{i}\left(t+dt\right)-\theta_{i}\left(t\right)=w\underset{j}{\sum }\left[\left(A_{ij}-k_{i}\delta_{ij}\right)\theta_{j}\left(t\right)\right]dt,$$

where *δ*_*ij*_ = 1 if *i* = *j*, and 0 otherwise. For d*t* reaching zero, the above equation gives

$$\frac{d\theta_{i}}{dt}=w\underset{j}{\sum }\left(A_{ij}-k_{i}\delta_{ij}\right)\theta_{j}.$$ (3)

Following Bollobás (1998), the discrete Laplacian of the graph, *L*, can be defined as *L* = *A* − *K*, where we use the matricial representation, *L*, for *L*_*ij*_ = *A*_*ij*_ − *k*_*i*_ *δ*_*ij*_. The diffusion dynamics on the graph, *G* = (*V*, *E*), can be written by following Eq. 3 as:

$$\theta^{\prime }\left(t\right)=wL\theta \left(t\right),$$ (4)

where ***θ*** is the vectorial representation of *θ*_*i*_, {***θ***}_*i*_ = *θ*_*i*_.

The most used expression for the network diffusion, Eq. 4, has an analytical solution:

$$\theta \left(t\right)=\exp\left(wLt\right)\theta \left(0\right).$$ (5)

In all the interesting cases, Eq. 5 is very hard to be computed exactly; its numerical solution is implemented in many software packages.

### 2.2 Reaction diffusion on networks

The diffusion dynamics on graphs can be used for inert substances (i.e., that do not increase, decrease, or react) when moving from nodes with a higher concentration to nodes with a lower concentration. It has many different applications, but when dealing with reactive substances or when the quantity of the substance is not preserved (it can be the quantity of proteins that aggregate in Alzheimer’s or Parkinson’s diseases or the progressive damage in traumatic brain injuries), the diffusion dynamics cannot be an adequate model to describe such phenomena.

A natural extension of Eq. 4 is the reaction–diffusion equation on a graph (Burioni et al., 2012)

$$\theta^{\prime }\left(t\right)=wL\theta \left(t\right)+f\left(\theta \left(t\right)\right).$$ (6)

The function ***f*** (***θ***) accounts both for the reaction of the substance inside nodes and the interaction between the nodes. In our case, we consider only the local reaction of the substances inside a node (which can be a model for protein accumulation inside a node or increased damage within a node), so the expression for the reaction function is {***f***}_*i*_(***θ*** (*t*)) = *rg*(*θ*_*i*_(*t*)), where *g*(*θ*) is the reaction term, which is the same for each node (homogeneity hypothesis of the reaction), and *r* is the reaction rate. Accordingly, simplifying Eq. 6, the reaction–diffusion equation on a graph studied in this work is represented as follows:

$$\theta_{i}^{\prime }=w\underset{j}{\sum }\left(A_{ij}-k_{i}\delta_{ij}\right)\theta_{j}+rg\left(\theta_{i}\right).$$ (7)

In general, there is no analytical solution to Eq. 7. Therefore, in order to study it, one must necessarily resort to numerical methods. See section “Numerical Integration” of the Supplementary Material for a simple numerical integration scheme for Eq. 7.

### 2.3 Reaction terms

For the reaction term *g*(*θ*), different choices can be made depending on the different kinds of reaction dynamics one wants to describe. One of the most widely used is the logistic growth function,

$$g\left(\theta \right)=\theta \left(1-\theta \right),$$ (8)

which can be found in many applications in different disciplines, for example, biology, medicine, physics, and chemistry. It was coupled for the first time to a diffusion operator in 1937, independently by Fisher and Kolmogorov, giving rise to the classical reaction–diffusion model (Fisher, 1937; Kolmogorov et al., 1937) often called the FKPP model.

$$\theta^{\prime }=w\nabla^{2}\theta +r\theta \left(1-\theta \right).$$

A brief discussion about the properties of the FKPP dynamics can be found in the section “Reaction–diffusion dynamics” of the Supplementary Material.

The logistic growth term takes into account both the exponential growth (with rate *r*) of the initial concentration of substances and a limiting factor that slows down the growth rate as the concentration approaches a maximum capacity. Without the loss of generality, usually, the limit concentration is fixed to *θ* = 1.

In some cases, at very small concentrations, the exponential growth induced by the logistic reaction term could be inappropriate. Thus, another very important reaction term, the Allee term (Allee, 1938; Stephens et al., 1999), is taken into account. It considers the presence of a concentration threshold before which the reaction is inhibited or not activated; at a concentration below the threshold, the *per capita* growth rate, i.e., $\dot{\theta }/\theta$ , is negative or zero. The Allee term is a valid model when, for small concentrations (small amounts of pathogen, inflammation, or population), the evolution dynamics fail to take hold. In the scientific literature, the Allee reaction term can be found in different forms (Courchamp et al., 2008) belonging to two main groups: the strong Allee effect, where the *per capita* growth rate is negative at low densities, and the weak Allee effect, where the reaction rate is positive at low densities but smaller than that at higher densities. In this work, we use two Allee reaction terms. The first is the classical analytical representation for the strong Allee effect (Courchamp et al., 1999).

$$g\left(\theta \right)=\theta \left(\theta -\theta_{c}\right)\left(1-\theta \right),$$ (9)

where *θ*_*c*_ is the threshold above (below) which the *per capita* growth rate is positive (negative), and the second (Vergni et al., 2012) lies in between the strong and weak Allee effects.

$$g\left(\theta \right)=\max\left\{\left(\theta -\theta_{c}\right)\left(1-\theta \right),0\right\}.$$ (10)

We call it the neutral Allee effect since, below the threshold, the growth rate is zero and the substance concentration remains constant (neglecting the diffusion term).

A detailed discussion of the different types of reaction terms introduced here, with associated illustrative figures, can be found in the section “Reaction dynamics” of the Supplementary Material.

Here, we want to stress that different reaction terms at varying initial conditions show very different phenomenology.

### 2.4 Data

We applied our model to a simple connectome constructed starting from the data provided by the B.A.T.M.A.N. tutorial (https://osf.io/fkyht/). The dataset was acquired from the University of Regensburg and is freely available upon request to the author. The acquired data and analysis pipeline are fully described in the tutorial. Here, we only summarize the main details and analysis steps.

All DWI images were acquired on a Siemens Prisma 3T MRI system equipped with a 64-channel receiver head coil and using a multi-shell acquisition scheme. Diffusion weighting of b = 1,000, 2,000, and 3,000 *s*/*mm* ^2^ was applied in 17, 31, and 50 directions, respectively. For each b-value, five images without diffusion-sensitizing gradients (i.e., “b0 images”) were acquired. Other DWI parameters are as follows: TR/TE = 8,500/110 ms; voxel size: 2.5 × 2.5 × 2.5 *mm* ^3^; matrix: 96 × 96; slices: 60.

Additionally, a high-resolution MPRAGE anatomical image dataset was acquired with the following parameters: FoV 256 × 256 mm, matrix size 256 × 256, 160 slices, 1 mm isotropic resolution, TR/TE = 1910/3.67 ms, flip angle 9^*o*^, and 5-min acquisition scheme.

Raw diffusion-weighted images were corrected for the eddy current, motion, and B0-field inhomogeneity using MRtrix3 software (Tournier et al., 2019). The anatomical T1-weighted images were linearly registered to the diffusion space using FSL (Smith et al., 2004). Constrained spherical deconvolution (CSD) and fiber orientation distribution (FOD) algorithms were used to reconstruct the tractograms. Then, spherical-deconvolution informed filtering of tractograms (SIFT) was used to decrease reconstruction biases and improve biological plausibility.

Cortical reconstruction and volumetric segmentation of anatomical data were performed with the FreeSurfer image analysis suite (Dale et al., 1999). To compute the structural connectome, the Human Connectome Project Multi-Modal Parcellation 1.0 (HCP MMP 1.0) (Glasser et al., 2016) was chosen.

In particular, the whole brain tractography and T1-based parcellations were combined to compute the atlas-based SC matrix in MRtrix. The nodes were represented by 379 distinct regions of interest (ROIs), and for each possible node pair, (*i*, *j*), interregional connectivity was defined as the number of reconstructed streamlines (NOS) scaled by the ROI volume, representing the edges of the connectome, *W*_*ij*_. The higher *W*_*ij*_ is, the stronger is the connection between the linked ROIs *i* and *j*.

Each ROI defines a vertex in the graph *G* = (*V*, *E*), and in order to obtain the adjacency matrix, *A*_*ij*_, which defines the set of edges, *E*, we use the weighted matrix *W*_*ij*_, introducing a threshold, *S*_*w*_, above (below) which the nodes (*i*, *j*) are connected (disconnected).

$$A_{ij}=\left\{\begin{array}{cc} 1 & \text{if }\;W_{\left(i,j\right)}\geq S_{w}\\ 0 & \text{if }\;W_{\left(i,j\right)}<S_{w} \end{array}\right.$$ (11)

In our case, we choose *S*_*w*_ = 0.034 in order to have a fully connected graph, i.e., from each node *i*, it is always possible to reach any other node of the graph. With this choice, there are 1,738 active links, and two nodes are particularly relevant, *i* = 53 (parcellation name L_3a), with a degree of 36 and an average shortest path to other nodes of 2.84, and *i* = 165 (parcellation name L_25), with a degree of 1 and an average shortest path to other nodes of 5.93. Those nodes are the most and the least connected ones, respectively.

## 3 Results

In this section, some evolution dynamics of Eq. 7 over a graph given by Eq. 11 will be presented at varying initial conditions and the reaction term, keeping all the parameters fixed, i.e., *r* = 1, *w* = 1, and *θ*_*c*_ = 0.001, in the presence of the Allee effect. In this way, it is possible to show how by only changing the nature of the reaction and the initial condition, while keeping all the parameters unchanged, the system can describe very different phenomenology associated with different outcomes of possible traumatic brain injuries or different development of degenerative brain diseases. The initial condition is defined as *θ*_*j*_(0) = *θ*_0_ *δ*_*ij*_, i.e., an empty network, except for a certain concentration, *θ*_0_, initially injected into a single node, *i*, which represents a particular brain region, or ROI, as discussed in Section 2.4.

A first quantity, able to characterize the evolution dynamics inside the connectome, is the percentage of the substance in the graph (Burioni et al., 2012) at a given time, *t*,

$$M\left(t\right)=\frac{1}{N}\underset{i\in V}{\sum }\theta_{i}\left(t\right),$$ (12)

where we recall that *N* is the total number of vertices. Since we are dealing with brain diseases or injuries, another important quantity is the number of nodes, i.e., regions in the brain, in which the concentration of disease or injury is above a given threshold:

$$N\left(t;\theta_{c}\right)=\underset{i\in V}{\sum }\Theta \left(\theta_{i}\left(t\right)-\theta_{c}\right),$$ (13)

where Θ(*x*) is the Heaviside step function with values 0 for *x* ≤ 0 and 1 for *x* > 0. In this work, we use *θ*_*c*_ = 0.001, which is the same value of the threshold for the Allee effect. The reason is simple: in the presence of the Allee effect, below the concentration threshold, the reaction dynamics are inhibited.

It is worth nothing that, with the simple network diffusion dynamics, Eq. 4, the quantity *M*(*t*) remains fixed, *M*(*t*) = *M*(0) = *θ*_0_/*N*, and the large time evolution of *N*(*t*; *θ*_*c*_) can be easily predicted as follows:

$$N\left(t\to \infty ;\theta_{c}\right)=\{\begin{array}{cc} N & \text{if }\;\;\;\theta_{0}/N>\theta_{c}\\ 0 & \text{if }\;\;\;\theta_{0}/N\leq \theta_{c} \end{array},$$

for each chosen initial condition since, at large times, the initial concentration, *θ*_0_, is spread over the entire connectome, and finally, a constant amount of concentration equal to *θ*_0_/*N* will be present in each node. A brief comparison between diffusion dynamics and reaction–diffusion dynamics can be found in the section “Reaction-diffusion dynamics” of the Supplementary Material.

**FIGURE 1:**
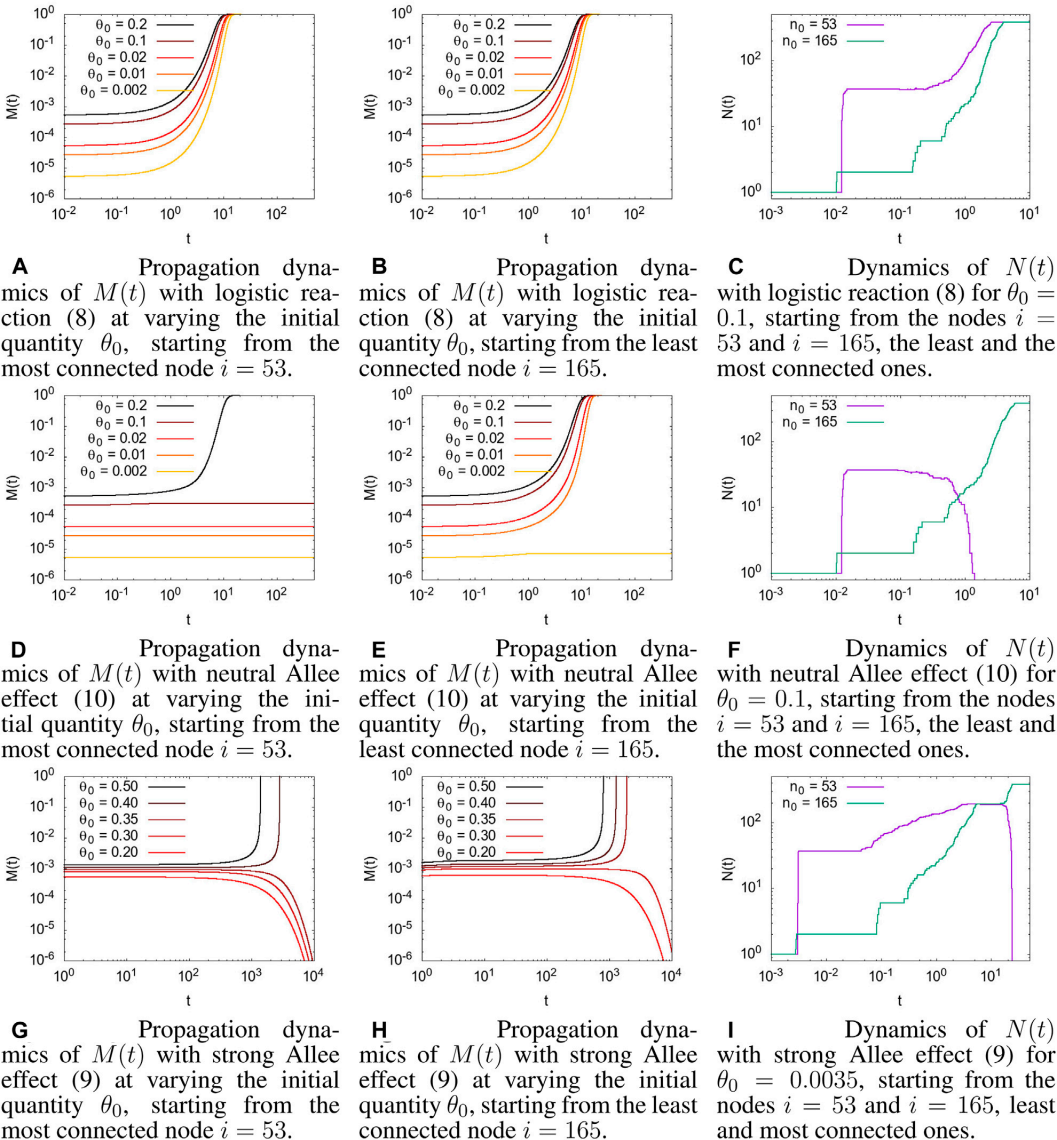
Comparison of different reaction dynamics, starting from a different initial node, *i*, and with different initial conditions *θ*(0) = *θ*_0_. *θ*_*c*_ = 0.001 for all the simulations with the Allee effect. **(A)** Propagation dynamics of *M*(*t*) (12) with a logistic reaction (8) on varying the initial quantity, *θ*_0_, starting from the most connected node *i* = 53. **(B)** Propagation dynamics of *M*(*t*) (12) with a logistic reaction (8) on varying the initial quantity, *θ*_0_, starting from the least connected node *i* = 165. **(C)** Dynamics of *N*(*t*) (13) with a logistic reaction (8) for *θ*_0_ = 0.1 starting from the nodes *i* = 53 and *i* = 165, the least and the most connected nodes. **(D)** Propagation dynamics of *M*(*t*) with a neutral Allee effect (10) on varying the initial quantity, *θ*_0_, starting from the most connected node *i* = 53. **(E)** Propagation dynamics of *M*(*t*) with a neutral Allee effect (10) on varying the initial quantity, *θ*_0_, starting from the least connected node *i* = 165. **(F)** Dynamics of *N*(*t*) with a neutral Allee effect (10) for *θ*_0_ = 0.1 starting from the nodes *i* = 53 and *i* = 165, the least and the most connected nodes. **(G)** Propagation dynamics of *M*(*t*) with a strong Allee effect (9) on varying the initial quantity, *θ*_0_, starting from the most connected node *i* = 53. **(H)** Propagation dynamics of *M*(*t*) with a strong Allee effect (9) on varying the initial quantity, *θ*_0_, starting from the least connected node *i* = 165. **(I)** Dynamics of *N*(*t*) with a strong Allee effect (9) for *θ*_0_ = 0.0035 starting from the nodes *i* = 53 and *i* = 165, the least and most connected nodes.

Instead, as shown in Figure 1, the variety in the concentration dynamics of Eq. 7, by changing the reaction term and initial conditions, is quite large, especially when compared to the behavior of the simple diffusion case.

In Figures 1A and B, the evolution of *M*(*t*) for the logistic reaction case, Eq. 8, for a different initial concentration, *θ*_0_, and two different initial nodes, *i* = 53 (Figure 1A) and *i* = 165 (Figures 1B), 1 is shown. As reported in Section 2.4, nodes *i* = 53 and *i* = 165 were chosen as the most and least connected node in the connectome, respectively. However, as discussed in Section 2.2, with the logistic reaction term, even a very little initial concentration was amplified by the initially exponential growth rate, and the dynamics always ended with the entire connectome full of concentration. The evolution dynamics of the number of nodes with a value of concentration higher than a given threshold, *N*(*t*; *θ*_*c*_), represented in Figure 1C, shows a different behavior when starting in the most connected node, *i* = 53, or in the least connected one, *i* = 165, denoting a different evolution dynamic inside the connectome; however, for large *t*, all the connectomes will be invaded, i.e., *M*(*t*) = 1 and *N*(*t*) = *N*.

The dynamics in the presence of the Allee effect is very different. Starting with the neutral Allee effect, Eq. 10, in Figures 1D and E, the propagation dynamics involve the whole connectome only when the initial concentration is above a given value that we call the *invasion value*. This value, which cannot be easily analytically computed, depends on the threshold value and, more interestingly, on the position in the network of the initial condition. When starting in the most connected node, *i* = 53, (Figure 1D) the invasion value appears to be higher than when starting in the least connected node, *i* = 165 (Figure 1E). This somehow counterintuitive behavior has a simple explanation: starting from the most connected site, the concentration invades most of the graph because of diffusion, which, meanwhile, causes the concentration to fall below the threshold since it has not had time to grow enough due to the reaction term, and the dynamic breaks down. On the other hand, starting from the least connected site, the reaction acts before diffusion, and when the concentration reaches enough nodes through diffusion, it has high values that allows it to survive and invade the entire graph. See Figure 1F for a graphical representation of the number of nodes that are above the threshold *θ*_*c*_.

A similar behavior is shown in Figures 1G and H, referred to as the strong Allee effect, with the presence of a minimal invasion concentration below which there is no invasion of the connectome. The most evident difference is in the spreading time of the concentration. The strong Allee effect has a negative growth rate for concentrations below the threshold that causes a slowdown in the evolution dynamics. The time taken to invade all the connectomes, when the initial concentration is above the invasion value, is much longer than that in the neutral Allee effect. Moreover, when the initial concentration is below the invasion value, the concentration in the connectome disappears (due to the negative growth rate); it also occurs if, initially, the concentration had invaded a good portion of the connectome (see Figure 1I). This may be consistent with a physiological situation, in which a brain injury heals itself or with the recovery from a brain disease.

**FIGURE 2:**
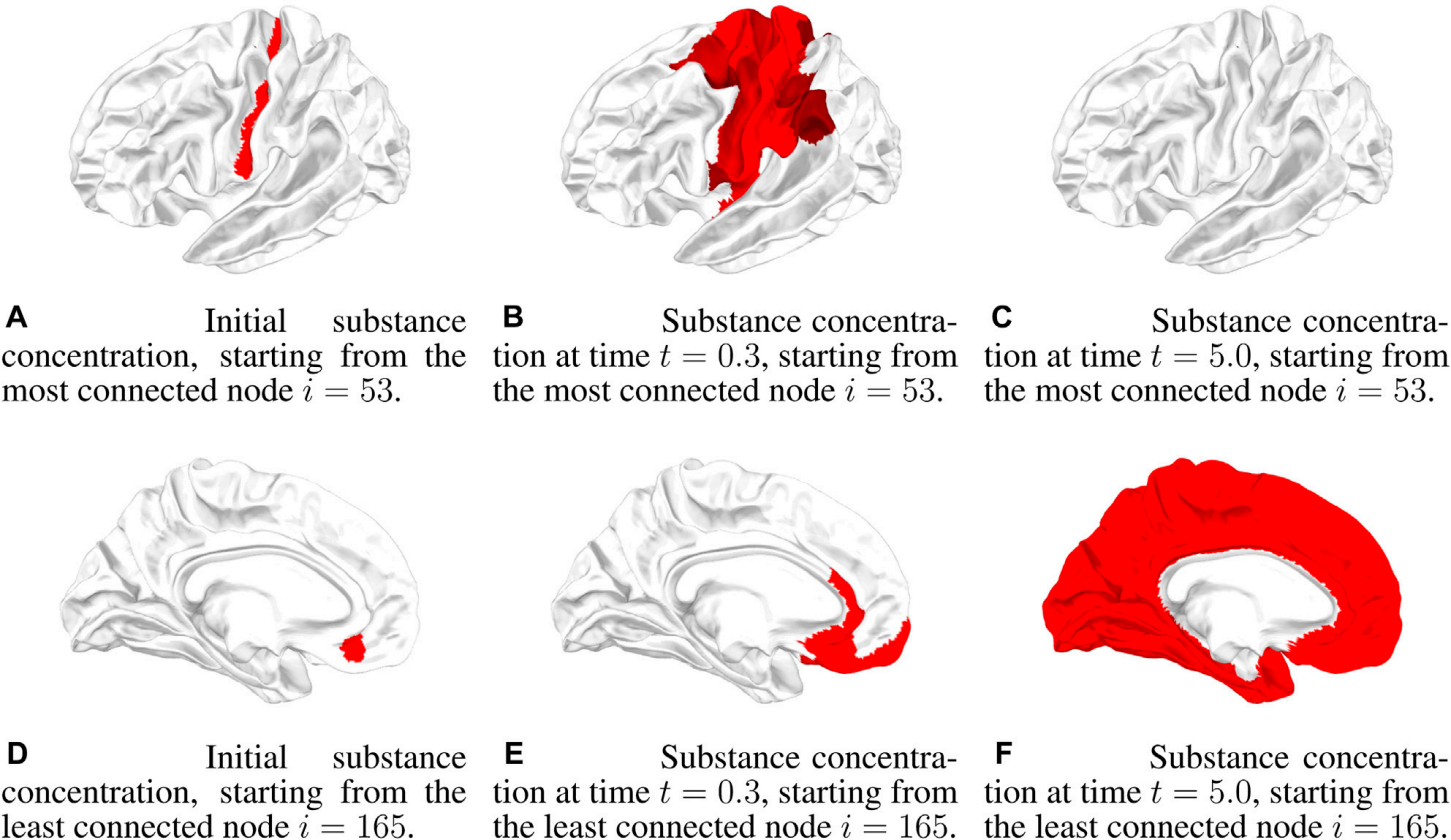
Three snapshots of the concentration dynamics in the brain at times *t* = 0.0, *t* = 0.3, and *t* = 5.0 in the case of a neutral Allee effect (10) for *θ*_0_ = 0.1. In the first row, the dynamics starts from the most connected node *i* = 53, while in the second row, the dynamics starts from the least connected node *i* = 165. **(A)** Initial substance concentration, starting from the most connected node *i* = 53. **(B)** Substance concentration at time *t* = 0.3, starting from the most connected node *i* = 53. **(C)** Substance concentration at time *t* = 5.0, starting from the most connected node *i* = 53. **(D)** Initial substance concentration, starting from the least connected node *i* = 165. **(E)** Substance concentration at time *t* = 0.3, starting from the least connected node *i* = 165. **(F)** Substance concentration at time *t* = 5.0, starting from the least connected node *i* = 165.

An example of a graphical representation of the propagation dynamics in the brain is given in Figure 2. Each node is a ROI of the brain, and the concentration in a node is pictorially represented by an intensity of red. Below the concentration threshold, the ROI is not colored, while above the concentration threshold, the intensity of red increases to saturation at a concentration given by 10*θ*_*c*_. The reaction dynamics and the initial conditions are the same as in Figure 1F, i.e., a neutral Allee effect is considered with *θ*_*c*_ = 0.001, and the initial condition is *θ*_0_ = 0.1, starting from two different nodes, the most connected and the least connected. We show three snapshots of the dynamics at *t* = 0, *t* = 0.3, and *t* = 5 (the time unit is arbitrary) for the two initial nodes. As discussed for Figure 1F, starting from the most connected node, there is an initial wide spread of concentration but with a low concentration value. Diffusion then brings the concentration below Allee’s threshold, and propagation is inhibited (see Figures 2A–C). Starting from the least connected node, the initial spread of concentration is not very wide, but, with a high concentration value, it continues to increase and propagates throughout the brain (see Figures 2D–F).

## 4 Discussion

Network diffusion models are able to describe the process of diffusion of substances within a medium whose structure can be schematized with a network, namely, a graph, where the nodes represent the basic elements of the medium, which can be, for example, the regions of the brain, and the edges represent the link between different nodes, for example, the structural or functional interactions between brain regions. A crucial feature of network diffusion models is that the substance in the medium is conserved as it spreads across the network. This means that the quantity *M*(*t*) is fixed, and asymptotically, the concentration of the substance in the network becomes constant, *θ*(*t* → *∞*) = *θ*_0_/*N*. This is a strong constraint that makes these models not realistic in many contexts, such as in cases of traumatic brain injuries or progressive brain diseases. On the other hand, reaction–diffusion models on networks are more flexible since they can describe the propagation of substances through the medium using different reaction dynamics, which can model different types of injury or disease evolution, without the constraint of the conservation of substances. As shown in the examples provided in the Results section, different choices of the reaction terms and the initial conditions (starting from the most or the least connected node) provide very different phenomenology, making these models highly flexible. In this work, we considered only the logistic non-linearity and Allee effects, which were usually used to describe the spreading of biological population and wave propagation. However, the reaction term can be modified by considering different nonlinear functions which could be more fitted for the description of different neurodegenerative diseases. Reaction–diffusion models comprise a huge variety of behaviors, and the choice of reaction terms must be guided by the phenomena of interest, which, in the case of neurodegenerative diseases, will be observed on different types of patient data. Although not presented in this work, a wide phenomenology can be observed by letting the reaction term and the other parameters of the model vary. We believe that any brain disease evolution can be described by a reaction–diffusion model using a particular reaction term, accurately calibrated model parameters, and a given initial condition, which could be, for example, the magnitude of the trauma in the case of a traumatic brain injury, or the extent of the disease in the case of a degenerative brain disease. In order to test the predictive ability of reaction–diffusion models for a specific brain disease, it is necessary to consider *ad hoc* experimental data (i.e., MRI or CT scans performed at different time intervals), which requires collaboration with hospital research centers and robust statistical methodology for calibrating the model parameters (both scalar, such as the reaction rate, diffusion rate, or the Allee threshold, and functional, such as the specific shape of the reaction term). All of this goes beyond the aim of this work, which is an important first step in introducing a complete modeling framework for studying the evolution of various progressive degenerative diseases and traumatic brain injuries.

Reliable models for brain pathologies are fundamental in the healthcare system. For traumatic brain injuries, these models could represent a supporting tool to medical staff, being instruments that provide predictions about the evolution of a trauma and, in turn, about the extent of the brain damage. In the examples shown in the Results section, we reported cases in which different positions of an initial trauma cause very different injury evolution. For degenerative brain diseases, as well as predicting the timing of disease progression, these models could help healthcare professionals to quantify the benefit of using a particular drug to treat a particular patient as drug use can be incorporated into the reaction model.

Finally, the network reaction–diffusion models proposed in this work can be extended by considering a weighted adjacency matrix and non-constant system parameters, allowing, for example, different reaction and diffusion rates in different brain regions. In terms of applications for brain pathologies, these models can consider a connectome with weighted links and variable reaction rates that take into account both the strength of the connection between brain regions and the different rates of progression of pathologies for a realistic description of brain diseases.

## Data Availability

The data analyzed in this study are subject to the following licenses/restrictions: the dataset is available upon request to the author of the B.A.T.M.A.N. package. Requests to access these datasets should be directed to https://osf.io/fkyht/.
