# Supplementary material for: On propagation in networks, promising models beyond network diffusion to describe degenerative brain diseases and traumatic brain injuries
Source: Front Pharmacol. 2024 Feb 26;15:1321171. doi: 10.3389/fphar.2024.1321171 (PMC10925667; doi:10.3389/fphar.2024.1321171)
Supplement: Supplementary file 1 [file Presentation1.pdf]

Supplementary Material

In this document, we want to discuss some known results about reaction models and reaction diffusion processes in order to present some insights in the theoretical basis of the main work. We also present a simple numerical integration method for the reaction diffusion equation on graphs (Eq. (7) of the main paper).

1 REACTION DYNAMICS

In this section, we consider in details the reaction terms considered in the main document, i.e., the logistic growth term

$$g(\theta) = \theta(1-\theta)\,, \tag{S1}$$

the neutral Allee effecct

$$g(\theta) = \max\{(\theta-\theta_c)(1-\theta), 0\}\,, \tag{S2}$$

and the strong Allee effect

$$g(\theta) = \theta(\theta-\theta_c)(1-\theta)\,. \tag{S3}$$

The evolution dynamics of the concentration of a given substance, θ, that undergoes a reaction equation, is given by the following differential equation

$$\begin{cases} \theta'(t) = rg(\theta(t)) \\ \theta(0) = \theta_0 \end{cases} \tag{S4}$$

where θ_0 is the initial concentration and r is the reaction rate. In the case of the exponential growth of a population due to a linear growth rate, $\dot{\theta} = r\theta$, r represents the constant per capita growth rate, $\dot{\theta}/\theta$, that is independent of the population size. Exponential growth is possible in nature only for short periods of time, after which population (or concentration) saturation occurs. Such a behaviour is modelled by the logistic growth (S1).

The differential equation (S4) can be analytically solved for each of the reaction terms presented, (S1), (S2) and (S3). Just as an example we present the solution in the case of the logistic term (S1).

$$\theta(t) = \frac{\theta_0}{\theta_0 + (1-\theta_0)e^{-rt}}\,. \tag{S5}$$

However, in this section, we want to qualitatively study the different dynamical behaviour given by Eq. (S4) occurring for the reaction terms here discussed and for different initial conditions.

Fig. 1a shows the logistic function (Eq. (S1), Eq. (8) in the main document), Fig. 1d the per capita growth rate, i.e. $\dot{\theta}/\theta$, and Fig. 1g the evolution dynamics of Eq. (S4) starting from different initial conditions, θ_0.

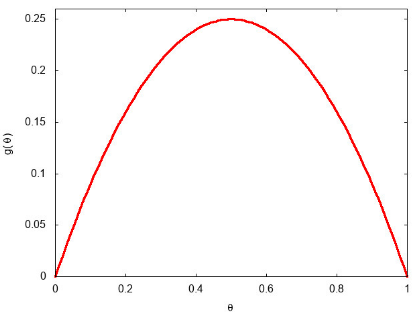

Figure 1a. Logistic function.

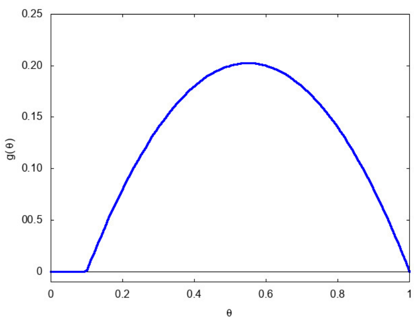

Figure 1b. Neutral Allee effect.

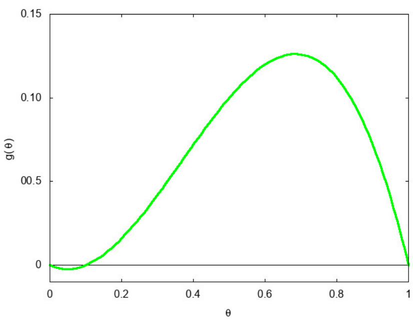

Figure 1c. Strong Allee effect.

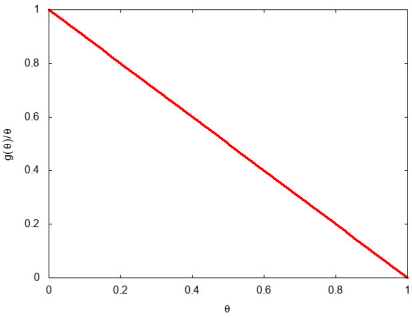

Figure 1d. Per capita growth rate for the logistic reaction term.

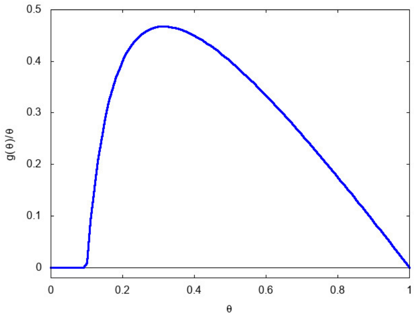

Figure 1e. Per capita growth rate for the neutral Allee effect.

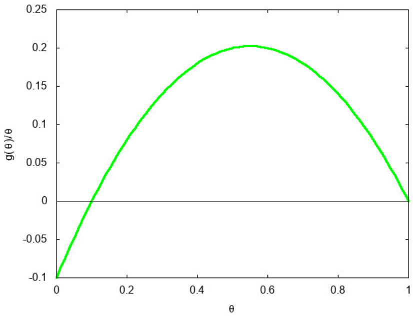

Figure 1f. Per capita growth rate for the strong Allee effect.

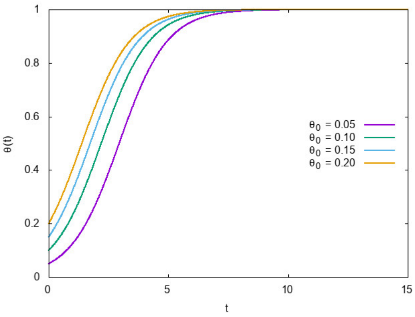

Figure 1g. Evolution dynamics for logistic reaction term for different initial conditions.

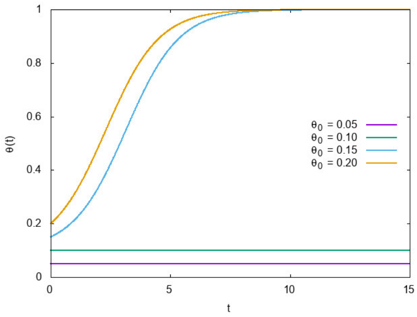

Figure 1h. Evolution dynamics for neutral Allee effect for different initial conditions.

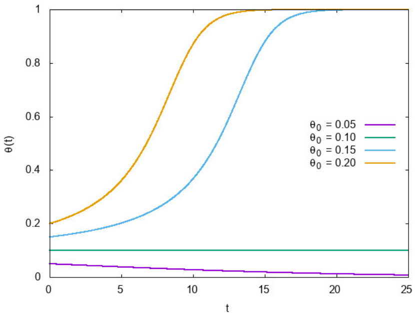

Figure 1i. Evolution dynamics for strong Allee effect for different initial conditions.

Figure 1. Reaction terms, $g(\theta)$, per capita growth rate, $\dot{\theta}/(\theta)$ and evolution dynamics (S4) for the reaction terms used in this section. In all the figures the concentration threshold is fixed $\theta_c = 0.1$. As for the evolution dynamics, we use different initial condition, $\theta(0) = \theta_0$, reported in the figures' labels.

Column 2 and 3 of Fig. 1 show analougous quantities for the neutral Allee effect (Eq. (S2), Eq. (10) in the main document) and the strong Allee effect (Eq. (S3), Eq. (9) in the main document), respectively.

It is important to emphasize the profoundly different behavior of the dynamics determined by the different reaction terms. The simple logistic term induces an initial exponential growth in the concentration leading to saturation at 1, regardless of the initial condition, that can be seen also in the analytical solution (S5). The neutral Allee effect show similar behaviour if the initial condition is above the threshold, $\theta_0 > \theta_c$, while for $\theta_0 \leq \theta_c$ there is no dynamics since $\theta'(t) = 0$ and $\theta(t) = \theta_0$. The strong Allee effect is the reaction term with the most peculiar dynamics, since the saturation time at 1 is much slower than in the other two cases, and, for initial conditions below θ_c, the per capita growth rate is negative and the concentration goes to zero.

Obviously, many different reaction terms can be introduced, capable of describing very different phenomenology, which may be useful for modelling different dynamics occurring in traumatic brain injuries or in progressive brain diseases.

2 REACTION-DIFFUSION DYNAMICS

In this section we discuss some known results about reaction diffusion process in one dimension and we compare the reaction diffusion dynamics with the simple diffusion dynamics.

The prototypical model for reaction-diffusion in one dimension is the non linear diffusion equation

$$\partial_t \theta(x,t) = w \partial_{xx} \theta(x,t) + r g(\theta(x,t)) \,, \tag{S6}$$

where the non-linear function, $g(\theta)$, could be one of the functions discussed in the above section.

In the case of the logistic non-linear term (S1), the reaction-diffusion equation is the one originally proposed in the seminal contribution by R. A. Fisher (Fisher, 1937) and A.N. Kolmogorov together with I.G. Petrovskii and N.S. Piskunov (Kolmogorov et al., 1937), which is commonly called FKPP equation. In the case of bounded initially conditions, i.e., when $\theta(x,0)$ is different from zero only in a bounded region, the FKPP equation shows travelling wave dynamics, i.e. $\theta(x,t) = \Theta(x - vt)$, that is a travelling wave of shape $\Theta(z)$ with speed

$$v = 2\sqrt{wr} \,. \tag{S7}$$

In the case of different reaction term, it is possible to determine upper and lower bound for the front speed (Aronson and Weinberger, 1978)

$$2\sqrt{wrg'(0)} \leq v \leq 2\sqrt{wr \sup_{\theta} \frac{g(\theta)}{\theta}} \,. \tag{S8}$$

Reaction terms for which $g'(0) = \sup_\theta g(\theta)/\theta$, i.e., the maximum per capita growth rate is in the origin (see Fig.s 1a and 1d), are called FKPP-like, and their front speed is that of the FKPP equation ((S7)). When the maximum per capita growth rate is not in zero (see Fig.s 1b and 1e) the dynamics are ruled by the region of $\theta(x,t)$ that are near the maximum per capita the growth rate.

The asymptotic evolution of the total quantity of substance (see Eq. (12) of the main document)

$$M(t) = \int_{-\infty}^{\infty} \theta(x,t) \mathrm{d}x \,, \tag{S9}$$

in the case of a propagating front, is given by

$$M(t) = M_o + 2vt \,. \tag{S10}$$

Examples of propagation dynamics in the case of FKPP-like dynamics (front propagation and total quantity of concentration in Fig.s 2a and 2b, respectively) and not FKPP-like dynamics (front propagation and total quantity of concentration in Fig.s 2a and 2b), can be found in Fig. 2, together with the case of

a simple diffusion dynamics. The comparison is very clear, a simple diffusion dynamic does not allow an increase in concentration and does not have true propagation, but simply a spreading of concentration. Propagation dynamics, on the other hand, are characterised by a moving front with a speed determined by the parameters of the system.

In the case of neutral or strong Allee effect, when the initial condition is below the concentration threeshold, or when diffusion time is faster than reaction time, it could be happen, also in the presence of a reaction diffusion dynamics, that front propagation may not take hold, and in this case we talk about quenching of the dynamics (see for example Fig.s 1d and 1g of the main paper).

3 NUMERICAL INTEGRATION

The diffusion equation on graph

$$\boldsymbol{\theta}'(t) = wL\boldsymbol{\theta}(t)\,, \tag{S11}$$

has an exact solution

$$\boldsymbol{\theta}(t) = \exp\left(wLt\right)\boldsymbol{\theta}(0)$$

that is almost never easy to calculate, so numerical computation methods are used. There are many software packages that calculate diffusion on graph. In the case where a reaction term is added, the exact analytical solution, in general, does not exist and one must necessarily resort to numerical computation methods, which, however, as far as we know, are not present in any software package. In this section we will present a simple numerical integration method that is based on the discretization of the time of the evolution dynamics.

Introducing a discrete integration time, Δt, the discrete-time version of the diffusion equation (S11) can be already found in Eq. (2) of the main paper, that we rearrange here as

$$\theta_i(t+\Delta t) = \theta_i(t) + w\left[\left(\sum_j A_{ij}\theta_j(t)\right) - k_i\theta_i(t)\right]\Delta t\,. \tag{S12}$$

In order to introduce the reaction term in the numerics, we consider a discrete solver, for time step Δt, of the simple reaction equation $\theta' = rg(\theta)$ as

$$G_{\Delta t}(\theta) = \theta + rg(\theta)\Delta t\,. \tag{S13}$$

Composing (S12) with (S13) we obtain an approximate solution in two steps for Eq. (7) of the main paper

$$\begin{cases} \theta_i^D(t+\Delta t) = \theta_i(t) + w\left[\left(\sum_j A_{ij}\theta_j(t)\right) - k_i\theta_i(t)\right]\Delta t \\ \theta_i(t+\Delta t) = G_{\Delta t}\left(\theta_i^D(t+\Delta t)\right) \end{cases} \tag{S14}$$

To guarantee the stability of the numerical solution are sufficient to fulfill the following conditions

$$\Delta t \ll \frac{2}{w \max_i k_i} \qquad \text{and} \qquad \Delta t \ll \frac{2}{r \max_\theta |g'(\theta)|}\,.$$

In the case of neutral Allee effect (S2), the function $|g'(\theta)|$ in $\theta = \theta_c$ is not defined. One must use $|g'_+(\theta_c)|$ as the right derivative of $g'(\theta)$ in θ_c.

REFERENCES

Aronson, D. G. and Weinberger, H. F. (1978). Multidimensional nonlinear diffusion arising in population genetics. *Advances in Mathematics* 30, 33–76

Fisher, R. A. (1937). The wave of advance of advantageous genes. *Annals of eugenics* 7, 355–369

Kolmogorov, A., Petrovskii, I., and Piskunov, N. (1937). Study of a diffusion equation that is related to the growth of a quality of matter, and its application to a biological problem. *Moscow University Mathematics Bulletin* 1

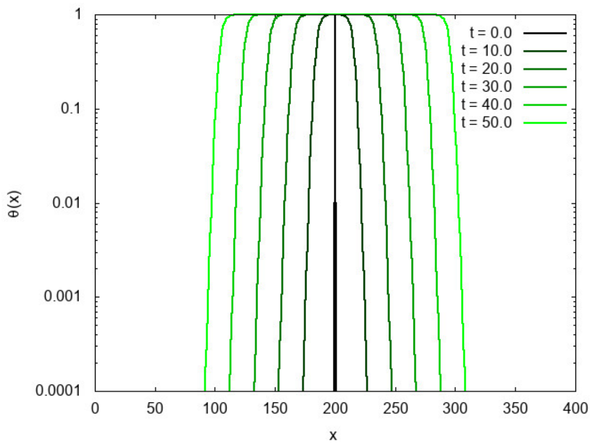

Figure 2a. Front evolution with logistic reaction dynamics (S1).

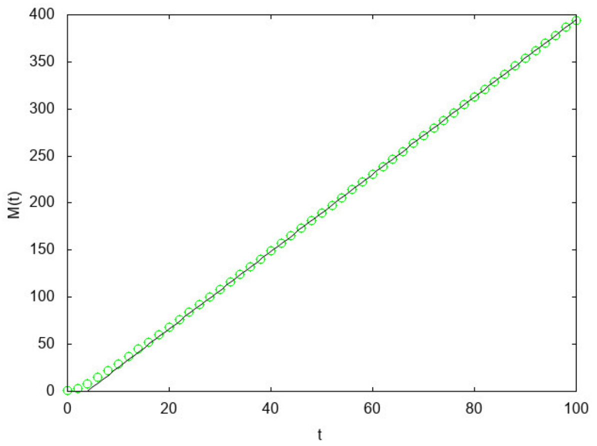

Figure 2b. Growth of concentration with logistic reaction dynamics.

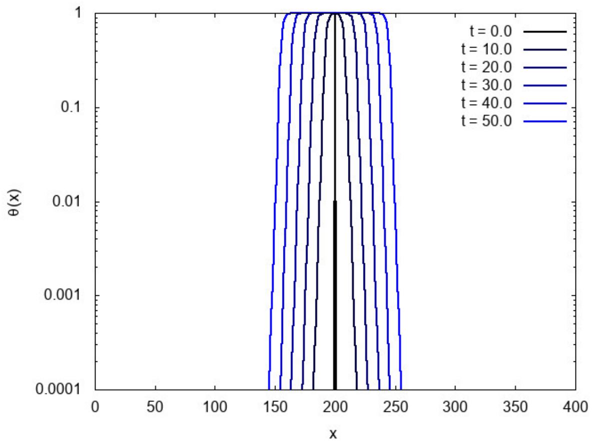

Figure 2c. Front evolution with neutral Allee effect (S2).

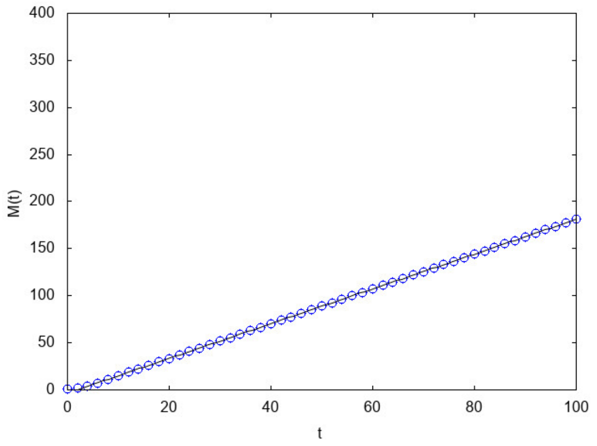

Figure 2d. Growth of concentration with neutral Allee effect.

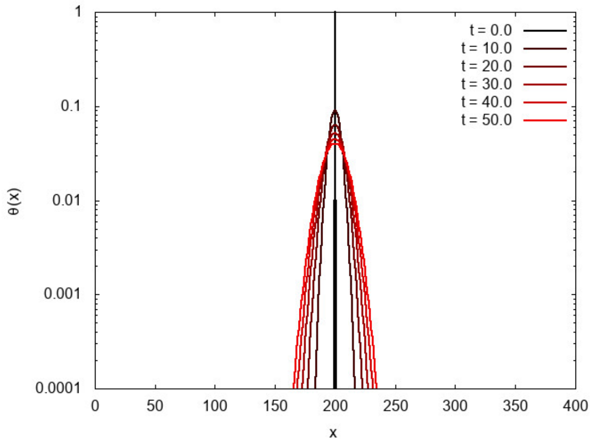

Figure 2e. Evolution of simple diffusion dynamics without reaction, i.e. $g(\theta(t)) = 0$.

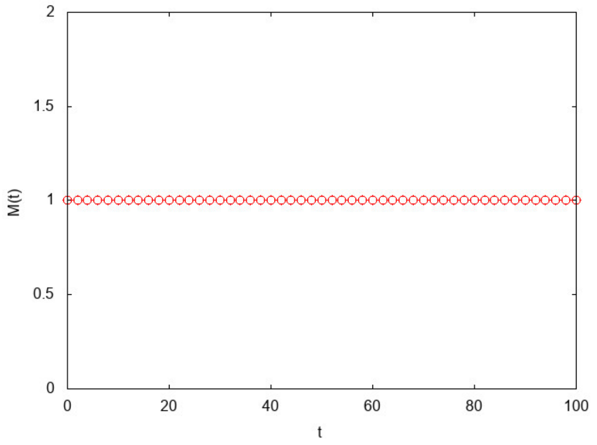

Figure 2f. Stationarity of concentration with diffusion dynamics.

Figure 2. Comparison of diffusion dynamics and reaction diffusion propagation dynamics. The initial condition is localized in $x = 200$ with a total concentration $M(0) = 1$.
